# Strain Engineering of Second-Harmonic Generation and Symmetry Breaking in Few-Layer ε-InSe

**DOI:** 10.3390/nano16150964

**Published:** 2026-08-06

**Authors:** Danliang Zhang, Sihan Liu, Peiran Li, Qing Ye, Ying Chen

**Affiliations:** 1 Key Laboratory of Low-Dimensional Quantum Structures and Quantum Control of Ministry of Education, Hunan Research Center of the Basic Discipline for Quantum Effects and Quantum Technologies, School of Physics and Electronics, Hunan Normal University, Changsha 410081, China; 202420112311@hunnu.edu.cn (S.L.); 202420112332@hunnu.edu.cn (P.L.); 2 State Key Laboratory of Pulsed Power Laser Technology, Key Laboratory of Electronic Restriction of Anhui Province, National University of Defense Technology, Advanced Laser Technology Laboratory of Anhui Province, Hefei 230037, China

**Keywords:** ε-InSe, Raman spectroscopy, second-harmonic generation, strain engineering

## Abstract

ε-phase indium selenide (ε-InSe), a non-centrosymmetric van der Waals layered semiconductor, exhibits broken inversion symmetry in all layer numbers, giving rise to exceptional second-order nonlinear optical responses and holding great promise for nonlinear optoelectronic applications. The dynamic control of the nonlinear efficiency of ε-InSe is crucial for its engineering applications. However, the quantitative manipulation of second-harmonic generation (SHG) intensity and crystal symmetry in few-layer ε-InSe via strain engineering is still lacking. In this work, we systematically investigate the modulation of SHG intensity and angle-resolved SHG patterns in few-layer ε-InSe under uniaxial tensile strain. Using a home-built straining apparatus, we apply controlled tensile strain and measure the strain-dependent SHG responses. The experimental results demonstrate that the SHG intensity of few-layer ε-InSe shows a non-monotonic response to increasing tensile strain, first increasing and then decreasing. Concurrently, the sixfold symmetry of the SHG pattern is broken, confirming the significant strain-induced modulation of the lattice symmetry. This study provides a viable route for the design of flexible and tunable nonlinear optoelectronic devices based on ε-InSe.

## 1. Introduction

Indium selenide (InSe) has attracted considerable attention owing to its superior properties, including ultrahigh carrier mobility [1,2,3,4] and layer-dependent bandgap [1,5,6,7]. It has been demonstrated as a promising candidate for high-efficiency photodetection [4,8,9,10], sliding ferroelectricity [11,12], and sensitive strain sensing [8,9,13]. InSe exhibits phases with different stacking sequences (β, γ, and ε) [14,15,16], among which the ε-phase InSe has a hexagonal unit cell and a non-centrosymmetric P-6m2 space group and is predicted to exhibit strong second-harmonic generation (SHG) nonlinear optical effects [14]. In stark contrast to 2H-phase transition metal dichalcogenides (TMDs) [17,18,19,20], ε-InSe retains broken inversion symmetry in all layer numbers and maintains phase-matched SHG conversion over thicknesses of several tens of nanometers [14], rendering it highly attractive for compact, ultrathin, and integrated nonlinear photonic devices.

Strain engineering is a powerful approach for tuning the optoelectronic properties of two-dimensional (2D) materials [21,22,23,24]. Owing to its extremely low Young’s modulus values and large elastic deformability [25], InSe is particularly susceptible to external strain while sustaining substantial deformation without fracture. The lattice and band structures of InSe have been shown to be sensitively modulated by uniaxial strain [13,26,27,28,29,30]. It is worth noting that SHG is highly sensitive to the strain tensor and exhibits a quantitative dependence on the crystal structure. SHG intensity can be exploited to map strain vector distributions [22], and angle-resolved SHG patterns further allow the determination of strain direction [31]. Experimental studies have shown that as uniaxial compressive strain increases, the SHG intensity in few-layer InSe decreases monotonically [32]. Moreover, pressure-dependent studies have demonstrated a reconfigurable structural symmetry transition from threefold rotational symmetry to mirror symmetry in layered InSe [29]. These studies confirm that strain significantly modulates the SHG response of InSe. However, a systematic investigation of the strain-dependent tuning of SHG intensity and crystal symmetry in few-layer ε-InSe is still lacking.

In this work, we present a systematic study of uniaxial tensile strain on mechanically exfoliated few-layer ε-InSe. Through strain-dependent SHG spectroscopy, we observe a non-monotonic evolution of the SHG intensity—initially increasing and subsequently decreasing with increasing tensile strain. SHG mapping reveals a strain-induced enhancement at small strain levels. Furthermore, by examining the angle-resolved SHG patterns under uniaxial tensile strain, we detect a discernible symmetry breaking of the SHG pattern. This study unveils an unconventional strain response in 2D ε-InSe and provides valuable insights for the development of flexible nonlinear optical devices based on 2D materials.

## 2. Materials and Methods

Few-layer InSe flakes mechanically exfoliated from high-quality ε-InSe single crystals (HQ Graphene, Groningen, the Netherlands) were transferred onto pre-cleaned 300 nm SiO_2_/Si substrates using a polydimethylsiloxane (PDMS)-assisted dry transfer method. The thickness of the exfoliated flakes was determined by atomic force microscopy (AFM; Bruker Multimode 8, Bruker Corporation, Billerica, MA, USA). The sample preparation process and representative AFM characterization of typical ε-InSe flakes are presented in Supplementary Figure S1.

For strain-dependent measurements, a polyvinyl alcohol (PVA) solution was spin-coated onto the InSe flakes and dried on a hot plate to remove residual moisture, ensuring firm adhesion between the PVA film and the flakes. Next, the PVA/InSe composite film was peeled off using tweezers and transferred to a transparent flexible polyethylene terephthalate (PET) substrate, with the two bonded together using a strong adhesive. Uniaxial strain was applied using a home-built apparatus, in which the PET substrate was bent by a precision micrometer head. Since the InSe flakes are encapsulated in PVA, the strain generated by bending the substrate is transferred uniformly and efficiently to the wafer, thus avoiding the formation of localized strain. The applied strain was calibrated from the bending geometry of the substrate and defined as *ε* = *τ* sin *θ*/2*a*, where *τ* is the substrate thickness, *θ* is the tangential angle at the minimum strain point, and 2*a* is the distance between the bending endpoints.

Raman and photoluminescence (PL) measurements were performed using a confocal Raman microscope (Witec, alpha-300, WITec GmbH, Ulm, Germany) with 532 nm and 488 nm continuous-wave laser excitation. The laser beam was focused onto the sample through a 50× objective (0.75 NA), providing a spot size of approximately 1 μm. The Raman and PL signals were collected in backscattering geometry through the same objective, dispersed by 1800 gr/mm or 150 gr/mm gratings, and recorded by a spectrometer equipped with a charge-coupled device (CCD). Raman spectroscopy analysis confirmed that each key sample used for SHG measurements was ε-InSe.

SHG measurements were conducted in reflection geometry using a femtosecond Ti–sapphire laser (Spectra-Physics, Santa Clara, CA, USA, pulse width ~100 fs, repetition rate 80 MHz). The fundamental beam at 800 nm passed through a 50:50 beam splitter and was focused onto the sample through a 50× objective. The backward-reflected SHG signal was collected by the same objective, filtered with a 400 nm bandpass filter to remove the fundamental beam, and detected by a spectrometer with a CCD. To ensure the accuracy of the tests, we repeated the SHG strain experiments multiple times and performed active refocusing and reference signal monitoring throughout the experiments.

For polarization-resolved SHG measurements, the polarization of the fundamental beam was controlled using a Glan–Thompson polarizer, and a rotating analyzer (WP25M-UB, Thorlabs, Newton, NJ, USA) placed in front of the detector was used to selectively measure SHG components parallel or perpendicular to the incident polarization. The SHG signal was recorded by rotating the sample over 360° in steps of 10°. Under unstressed and stressed conditions, the fitted curves follow the relationships *I* ∝ *I*_0_ cos^2^ (3*θ*) and *I* ∝ 1/4[*A* cos (3*θ*) + *B* cos (2*φ* + *θ*)]^2^, respectively. The parameters defined here are as follows: *I* is the intensity of polarization-dependent SHG; *A* is a coefficient related to the unstrained SHG intensity and the isotropic strain-induced contribution; *B* is a coefficient describing the anisotropic strain-induced contribution; *θ* is the angle between the polarization direction of the incident light and the armchair direction of the crystal; and *φ* is the angle between the strain direction and the armchair direction.

## 3. Results and Discussion

**Figure 1:**
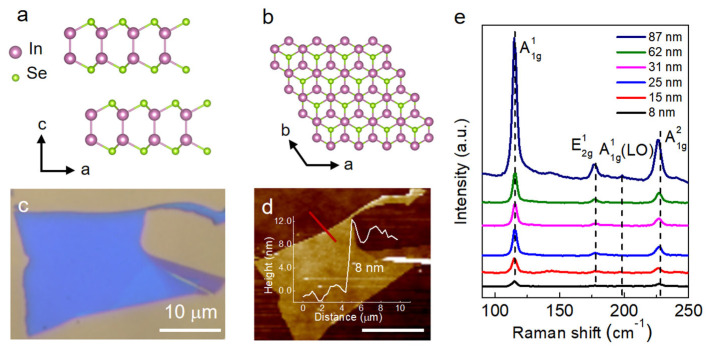
AFM and Raman characterization of ε-InSe: (**a**,**b**) Side-view (**a**) and top-view (**b**) schematic illustrations of the typical atomic structure of bilayer ε-InSe. Purple and green spheres represent indium (In) and selenium (Se) atoms, respectively. (**c**) Optical microscopy image of a representative ε-InSe flake on a SiO_2_/Si substrate. (**d**) Corresponding AFM topography images; the inset shows the height profile along the red line. All scale bars are 10 μm. (**e**) Raman spectra of ε-InSe flakes with different thicknesses.

Figure 1a,b present side-view and top-view schematic illustrations of the ε-InSe crystal structure. The basic structural unit consists of Se–In–In–Se atomic layers stacked in a non-centrosymmetric manner, ensuring broken inversion symmetry in all layer numbers. We prepared few-layer ε-InSe flakes by mechanical exfoliation. The optical micrograph in Figure 1c displays clear edges and a flat morphology. The corresponding atomic force microscopy (AFM) topography image (Figure 1d) shows that the surface is smooth and wrinkle-free. The height profile measured along the red dashed line (inset in Figure 1d) confirms that the layer thickness is approximately 8 nm, equivalent to about 10 layers, providing a high-quality research platform for subsequent studies based on thickness-dependent SHG and strain modulation.

Raman spectroscopy is a sensitive probe of the thickness-dependent lattice dynamics in 2D InSe. Figure 1e displays Raman spectra of InSe flakes with thicknesses ranging from 8 nm to 87 nm. With decreasing thickness, the intensity ratios of the different Raman modes exhibit pronounced variations. Three prominent vibrational peaks are observed at approximately 114.4, 143, and 226.5 cm^−1^, corresponding to the $A_{1g}^{1}$, $E_{2g}^{1}$, and $A_{1g}^{2}$ vibration modes, respectively. A Raman peak at approximately 198.5 cm^−1^ is clearly resolved in thicker flakes, and its intensity exhibits an increase with thickness, in good agreement with earlier studies [14], thereby corroborating the ε-phase nature of the InSe crystals. Furthermore, the PL spectra of InSe show a clear thickness dependence (Supplementary Figure S2), with a systematic blueshift of the bandgap as the thickness decreases, arising from quantum confinement.

**Figure 2:**
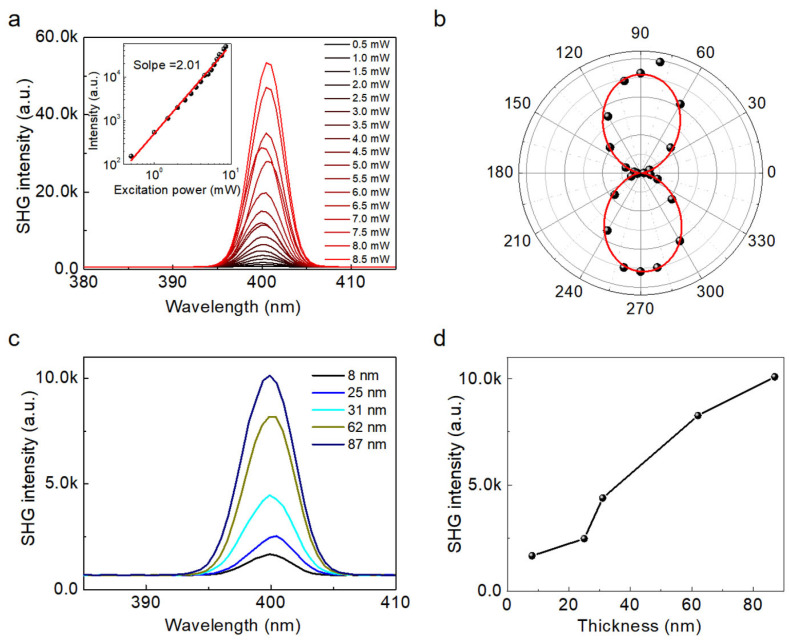
Power- and thickness-dependent SHG spectra of ε-InSe: (**a**) SHG intensity as a function of excitation power. The curve evolves from black to red, indicating a gradual increase in excitation power. Inset: double-logarithmic plot of SHG intensity versus excitation power; the red line is the linear fit with a slope of ~2.01. (**b**) Polar plot of the linear polarization response of the detected SHG. The red curve shows the fitted plot of the SHG signal. (**c**) SHG spectra of ε-InSe flakes with different thicknesses. (**d**) SHG intensity extracted from (**c**) as a function of flake thickness.

SHG provides a non-destructive and sensitive means of probing the nonlinear optical properties and crystal symmetry of 2D InSe. We first performed power-dependent SHG measurements on ε-InSe (Figure 2a). The extracted SHG intensity plotted on a double-logarithmic scale (inset of Figure 2a) yields a linear fit with a slope of approximately 2.01, unambiguously confirming that the detected signal originates from a second-order nonlinear process. To determine the polarization characteristics of the SHG response, we fixed the excitation polarization and rotated the detection polarizer. The resulting SHG intensity exhibits a clear linear polarization response (Figure 2b), indicating that the second-harmonic polarization field has a well-defined vibrational direction.

Figure 2c shows SHG spectra of ε-InSe flakes with different thicknesses under identical excitation conditions. The SHG intensity increases markedly with increasing thickness from 8 nm to 87 nm. The thickness-dependent SHG intensity of ε-InSe, extracted from the spectrum, exhibits a stepwise increase (Figure 2d). The deviation of the SHG intensity from a quadratic power dependence on thickness is likely caused by strong band-edge absorption, coherence length limitations, multilayer interference, and substrate effects. Importantly, ε-InSe exhibits SHG activity for all layer numbers, without the even-odd layer oscillation characteristic of 2H-phase TMDs [18,33,34], owing to its non-centrosymmetric crystal structure, which maintains inversion symmetry breaking in all layers. Our experimental results show that the second-order nonlinear coefficient χ^(2)^ of ε-InSe is approximately 25 times that of LiNbO_3_ with the same thickness and also exceeds that of monolayer TMDs systems [35,36,37] (Supplementary Figure S3 and Table S1), confirming ε-InSe as an outstanding 2D nonlinear optical material.

**Figure 3:**
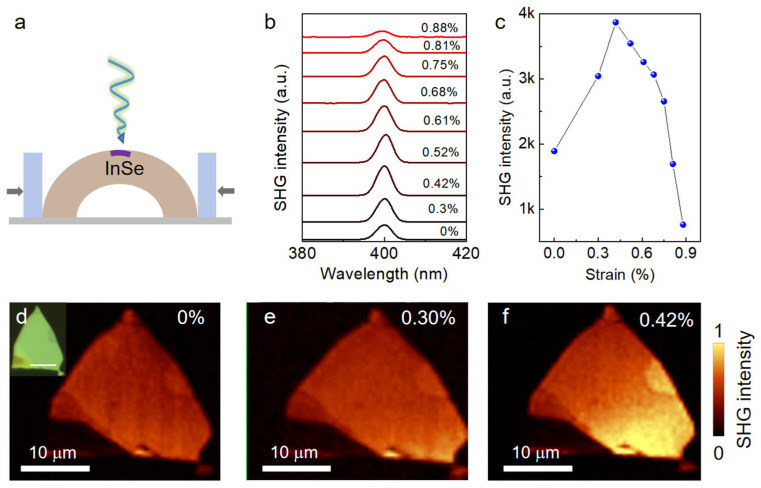
Strain tuning of the SHG of ε-InSe: (**a**) Schematic illustration of the strain-tuning setup. The curved line with an arrow represents the incident excitation light. (**b**) Evolution of SHG spectra of few-layer ε-InSe with increasing tensile strain. (**c**) Extracted SHG intensity as a function of applied strain. The SHG spectra were fitted using a Gaussian line shape to extract the peak intensities. The standard deviation of each fit is approximately 0.005, and the goodness of fit is evaluated, with R^2^ values exceeding 0.99. (**d**–**f**) Normalized SHG intensity mapping at strains of 0%, 0.30%, and 0.42%, respectively. The insets show optical images of the ε-InSe sample.

To explore the influence of strain on the crystal structure of InSe, we measured the strain-dependent SHG spectra. Experimentally, the PVA-encapsulated few-layer ε-InSe flake (about 25 nm) on the flexible substrate was mounted on the home-built uniaxial tensile straining apparatus. Because the PVA layer forms strong adhesion with the ε-InSe flake, bending the substrate by displacing the two ends effectively transfers strain to the flake (Figure 3a). We observed a non-monotonic evolution of the SHG intensity with increasing tensile strain (Figure 3b). For tensile strains up to 0.42%, the SHG intensity increases by more than a factor of two. Beyond 0.42%, the SHG intensity gradually decreases with further increasing strain. This strain-dependent behavior is tentatively attributed to small strains inducing subtle lattice-constant changes that modify the electronic band structure and transition matrix elements, enhancing the effective second-order nonlinear susceptibility χ^(2)^ and thereby strengthening the optical resonance associated with SHG [38,39,40]. Theoretical calculations of the strain-dependent evolution of the band structure in ε-InSe and its effect on χ^(2)^ are topics that require further investigation in the future. At larger strains, the band structure undergoes more substantial reconstruction, leading to reduced transition matrix elements and consequent quenching of the SHG signal [41]. The observed repeatability and cyclic stability confirm the intrinsic nature of the non-monotonic strain-tuning behavior in ε-InSe and validate its potential for use in reversible and reusable flexible optoelectronic applications (Supplementary Figures S4 and S5).

To verify the uniformity of the applied strain, we performed SHG spatial mapping of the ε-InSe flake. Under strain, the SHG signal exhibits uniform distribution over the entire flake region (Figure 3d–f). At lower tensile strains (0–0.42%), a general increase in SHG intensity was observed throughout the flake structure. At higher strains, however, excessive bending leads to focal drift, preventing complete SHG mapping of the sample. SHG mapping of the other ε-InSe samples also showed a similar evolution trend, confirming the efficient and uniform transfer of strain to the flake (Supplementary Figure S6). The PL spectra of few-layer ε-InSe also display a clear strain dependence (Supplementary Figure S7), corroborating the effective strain modulation of the crystal structure. These findings highlight the potential of strain engineering for tunable nonlinear optoelectronic devices and strain sensors based on ε-InSe.

**Figure 4:**
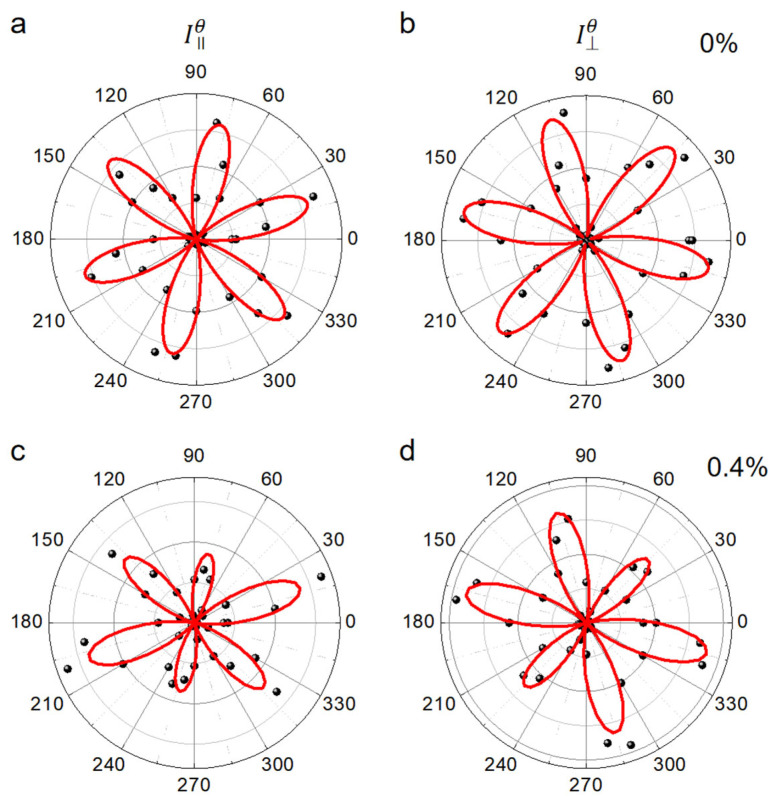
Polarization-resolved SHG in ε-InSe under conditions without and with strain: (**a**,**b**) Parallel (**a**) and perpendicular (**b**) polarization angle-resolved SHG patterns of few-layer ε-InSe under unstrained conditions, showing sixfold symmetric patterns. (**c**,**d**) Corresponding patterns under 0.4% uniaxial tensile strain, showing broken sixfold symmetry and asymmetric intensity distribution. Solid black circles represent experimental data. The red curve shows the results of the fit.

We next explored the use of SHG for strain sensing in few-layer ε-InSe. Polarization-resolved SHG is sensitive to lattice symmetry, as the SHG intensity is directly related to the second-order nonlinear susceptibility tensor χ^(2)^. Strain modifies the lattice structure and consequently alters χ^(2)^, breaking the symmetry of the SHG polar patterns. Under unstrained conditions, the parallel $I_{∥}^{\theta }$ polarization (Figure 4a) and perpendicular $I_{⊥}^{\theta }$ polarization (Figure 4b) angle-resolved SHG patterns of ε-InSe both exhibit well-defined sixfold patterns, reflecting the $D_{3h}^{1}$ symmetry and threefold rotational symmetry of the ε-InSe lattice [14]. When a tensile strain of 0.4% is applied, the sixfold symmetry of both parallel (Figure 4c) and perpendicular (Figure 4d) polarization SHG patterns is clearly broken, and the petal intensities become markedly asymmetric [22,31]. This symmetry breaking arises from the uniaxial strain-induced reduction in the in-plane rotational symmetry, which differentially modifies the symmetry-equivalent components of the nonlinear susceptibility tensor. These results demonstrate that the symmetry change of the angle-resolved SHG patterns is highly sensitive to strain, providing an effective optical means for determining both the magnitude and direction of strain in ε-InSe.

## 4. Conclusions

In summary, we have systematically investigated the modulation of angle-resolved second-harmonic generation in few-layer ε-InSe by uniaxial tensile strain. ε-InSe exhibits a large second-order nonlinear coefficient and maintains SHG activity for all layer numbers. Under uniaxial tensile strain, the SHG intensity shows a non-monotonic evolution, initially increasing and then decreasing. This is attributed to strain-induced modifications of the band structure and transition matrix elements, which in turn affect the second-order nonlinear susceptibility. Under unstrained conditions, the SHG polar patterns display sixfold symmetry, which becomes clearly broken under tensile strain, reflecting the strain-induced reduction in in-plane rotational symmetry and providing an optical criterion for identifying strain direction. This work demonstrates the potential of strain engineering for optimizing the nonlinear optical performance of ε-InSe and lays a foundation for its application in flexible tunable nonlinear optoelectronic devices and strain sensing.

## Data Availability

The original contributions presented in this study are included in the article/Supplementary Materials. Further inquiries can be directed to the corresponding authors.

## Supplementary Materials

The following supporting information can be downloaded at https://www.mdpi.com/article/10.3390/nano16150964/s1, Figure S1: Preparation and AFM characterization of ε-InSe. Figure S2: Thickness-dependent PL spectra of ε-InSe. Figure S3: Comparison of second-order nonlinear coefficients χ^(2)^. Table S1. Comparison of χ^(2)^ of 2D semiconductors. Figure S4: Reproducibility of the strain-dependent SHG modulation in few-layer ε-InSe. Figure S5: Cyclic loading test of strain-dependent SHG in few-layer ε-InSe. Figure S6: Spatial mapping of strain-dependent SHG in few-layer ε-InSe. Figure S7: Strain-dependent PL spectra of ε-InSe. Reference [42] is cited in the supplementary materials.
