# Supplementary material for: Strain Engineering of Second-Harmonic Generation and Symmetry Breaking in Few-Layer ε-InSe
Source: Nanomaterials (Basel). 2026 Aug 6;16(15):964. doi: 10.3390/nano16150964 (PMC13467642; doi:10.3390/nano16150964)
Supplement: Supplementary file 1 [file nanomaterials-16-00964-s001.zip › nanomaterials-4460778-supplementary.pdf]

Supplementary Information

Strain Engineering of Second-Harmonic Generation and Symmetry Breaking in Few-Layer ε-InSe

Danliang Zhang[1], Sihan Liu[1], Peiran Li[1], Qing Ye[2]*, Ying Chen[2]**

[1]Key Laboratory of Low-Dimensional Quantum Structures and Quantum Control of Ministry of Education, Hunan Research Center of the Basic Discipline for Quantum Effects and Quantum Technologies, School of Physics and Electronics, Hunan Normal University, Changsha 410081, China.

**Email: danliangzhang@hunnu.edu.cn*

[2]State Key Laboratory of Pulsed Power Laser Technology, Key Laboratory of Electronic Restriction of Anhui Province, National University of Defense Technology, Advanced Laser Technology Laboratory of Anhui Province, Hefei 230037, P. R. China.

Email: yeqing18@nudt.edu.cn; chen_ying@hnu.edu.cn

Table of Contents:

Note 1. Sample Preparation and AFM Characterization

Note 2. Thickness-Dependent PL Spectroscopy

Note 3. Determination of the Second-Order Nonlinear Coefficient $\chi^{(2)}$ of ε-InSe

Note 4. Measurements of the Reproducibility and Cyclability of SHG Strain Engineering in ε-InSe

Note 5. Strain-Tunable PL spectroscopy

Note 1. Sample Preparation and AFM Characterization

Figure S1a illustrates the PDMS-assisted dry transfer process for transferring mechanically exfoliated ε-InSe flakes onto a SiO_2/Si substrate. This transfer method effectively avoids organic solvent contamination, ensuring a clean sample surface. The typical pattern of a few-layer ε-InSe flake exhibits straight and well-defined edges with lateral dimensions of approximately 30–50 μm (Figure S1b), satisfying the requirements for optical characterization. To confirm the thickness of the ε-InSe flakes, AFM measurements were systematically performed. Figure S1c,d present AFM topography images of flakes with different thicknesses and the corresponding height profiles along the red lines. The measured thicknesses are approximately 25 nm, 31 nm, and 87 nm, corresponding to about 31, 39, and 109 layers (assuming a monolayer thickness of ~0.8 nm). The flakes exhibit smooth, wrinkle-free surfaces and uniform morphology. The flat morphology and precisely calibrated thickness provide a reliable sample platform for subsequent strain-dependent SHG experiments.

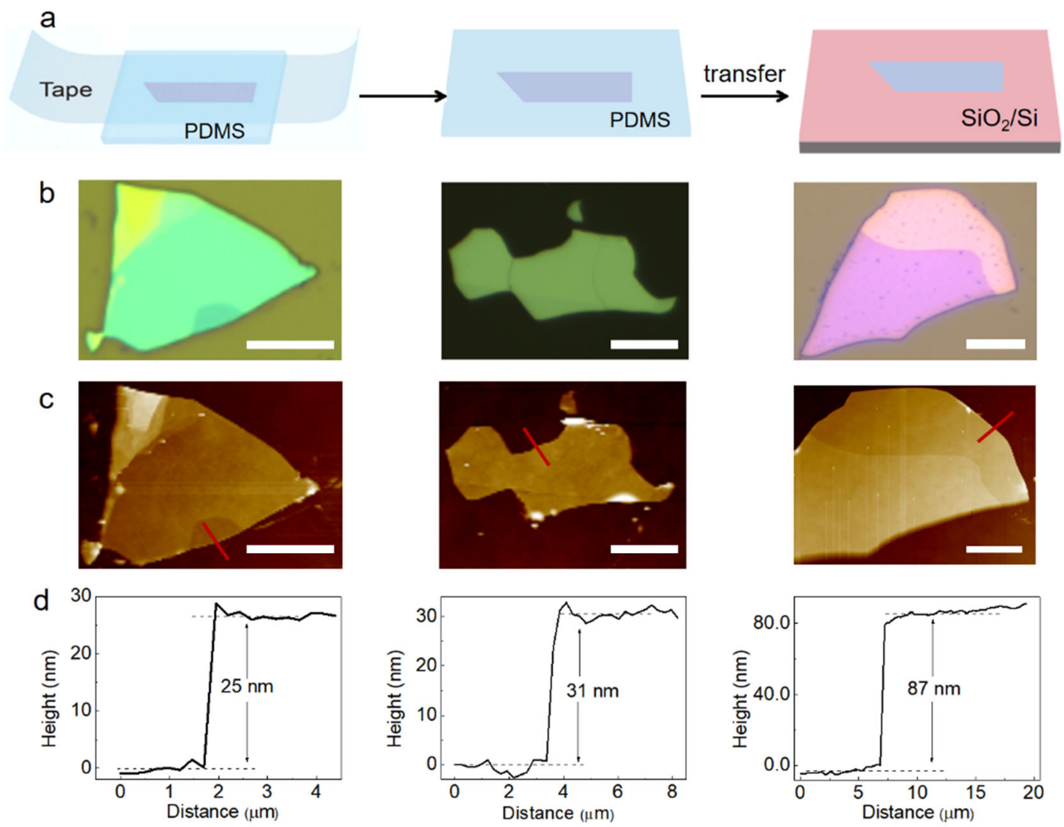

Figure S1. Preparation and AFM characterization of ε-InSe: (a) Schematic illustration of the PDMS-assisted dry transfer process for transferring mechanically exfoliated ε-InSe flakes onto a SiO_2/Si substrate. (b) Optical microscopy image of a representative few-layer ε-InSe flake after transfer. (c) AFM topography images of flakes with different thicknesses and (d) corresponding height profiles along the red lines. All scale bars are 10 μm.

Note 2. Thickness-Dependent PL Spectroscopy

We performed PL spectroscopy on ε-InSe flakes with representative thicknesses to analyze the thickness-dependent bandgap (Figure S2). As the thickness increases from 5 nm to 12 nm, the emission peak exhibits a pronounced redshift from 1.43 eV to 1.27 eV. For thicknesses ranging from 12 nm to 87 nm, the emission energy gradually redshifts to approximately 1.25 eV. The observed PL peak shift is attributed to the thickness-dependent reduction of the bandgap. Concurrently, the PL emission intensity increases by approximately a factor of four as the thickness increases from 5 nm to 87 nm. This enhancement in PL intensity with increasing thickness is likely associated with the weakening of quantum confinement effects and the reduction in the relative proportion of defect states [1, 2].

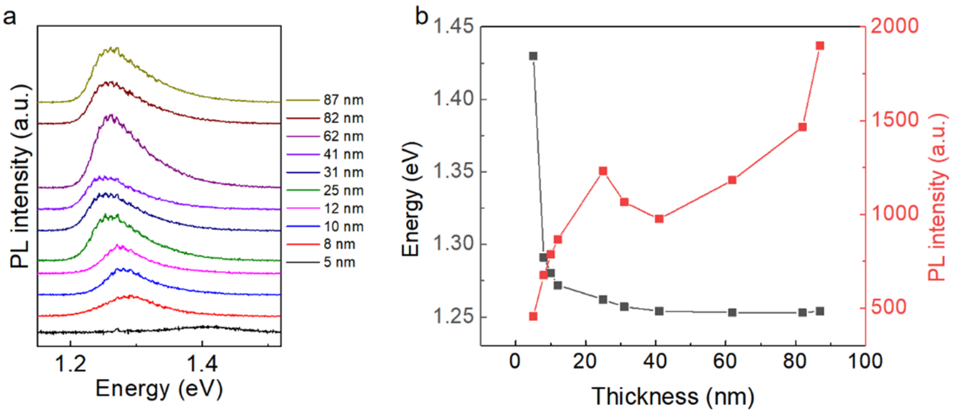

Figure S2. Thickness-dependent PL spectra of ε-InSe: (a) Room-temperature PL spectra of ε-InSe flakes with different thicknesses. (b) Extracted peak energy and PL intensity as a function of flake thickness.

Note 3. Determination of the Second-Order Nonlinear Coefficient $\chi^{(2)}$ of ε-InSe

To quantitatively determine the second-order nonlinear coefficient $\chi^{(2)}$ of ε-InSe, we measured and compared the SHG intensities of bulk $LiNbO_3$ crystals, monolayer WS_2, and few-layer ε-InSe under identical experimental conditions (Figure S3a). A commercial thin $LiNbO_3$ crystal was used as a reference sample to estimate the $\chi^{(2)}$ values of monolayer WS_2 and few-layer ε-InSe. The effective $\chi^{(2)}$ was calculated using the following relationship: $\frac{\chi^2{}_{eff(LiNbO3)}}{\chi^2{}_{eff(InSe)}} = \frac{L_{InSe}}{L_{LiNbO3}}\frac{P_{in(LiNbO3)}}{P_{in(InSe)}}\sqrt{\frac{I_{out(LiNbO3)}}{I_{out(InSe)}}}$. Here, L is the sample length, P_{in} is the incident laser power, and I_{out} is the emitted SHG intensity. The reference $\chi^{(2)}$ value of $LiNbO_3$ was taken as 0.0318 nm/V [3], with a thickness of 600 nm. The thicknesses of monolayer WS_2 and few-layer ε-InSe are approximately 0.7 nm and 20 nm, respectively. The calculated second-order nonlinear coefficients are presented in Figure S3b. The $\chi^{(2)}$ of ε-InSe reaches 0.786 nm/V, which is approximately 1.7 times that of WS_2 (0.452 nm/V). We also acknowledge that a more precise determination of the absolute $\chi^{(2)}$ value would require a more rigorous theoretical model (e.g., accounting for Fresnel coefficients, interference, and absorption) in future work.

a

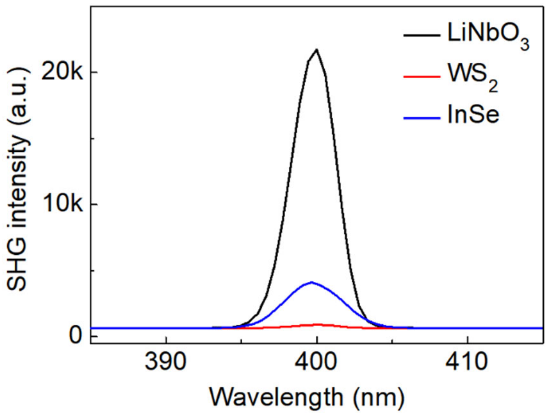

b

Materials	$LiNbO_3$	WS_2	InSe
$(\chi^2)/(nmV^{-1})$	0.0318	0.452	0.786

Figure S3. Comparison of second-order nonlinear coefficients $\chi^{(2)}$: (a) Comparison of SHG spectra from $LiNbO_3$ crystal, monolayer WS_2, and ε-InSe. (b) Extracted second-order nonlinear coefficients $\chi^{(2)}$ of the three materials based on the measured SHG intensities.

Table S1 summarizes the second-order nonlinear coefficients $\chi^{(2)}$ of InSe from previous reports, along with those of WS_2, MoS_2, and GaSe for comparison. InSe shows a $\chi^{(2)}$ of 6.39 nmV^{-1}, greatly exceeding monolayer WS_2 (0.5 nmV^{-1}) and MoS_2 (0.25 nmV^{-1}) and surpassing even GaSe (2.4 nmV^{-1}), which is well known for its strong nonlinearity. These results highlight the superior second-order nonlinear optical response of InSe, positioning it as a highly promising 2D material for nonlinear optics.

Table S1. Comparison of $\chi^{(2)}$ of 2D semiconductors.

	$\chi^{(2)}$ (nmV^{-1})	λ_{exc} (nm)	Thickness	Referenc
InSe	6.39	800	1L	4
WS_2	0.5	832	1L	5
MoS_2	0.25	870	1L	6
GaSe	2.4	1210	1L	7

Note 4. Measurements of the Reproducibility and Cyclability of SHG Strain Engineering in ε-InSe

We performed uniaxial tensile strain-dependent SHG measurements on three few-layer ε-InSe samples, which consistently exhibited highly reproducible behavior. As shown in Fig. S4, the SHG intensity in all three samples displays a non-monotonic evolution with increasing tensile strain, initially increasing and then decreasing, with the peak enhancement consistently observed at a strain of approximately 0.42%. During the straining process, the SHG spectral peak positions remained essentially unchanged across all samples, indicating that strain did not induce significant phase mismatch or drastic band structure reconstruction. The excellent agreement among the three independent samples in both the SHG intensity evolution trend and the strain value at which peak enhancement occurs demonstrates that the observed non-monotonic strain-tuning behavior is an intrinsic property of ε-InSe and confirms the high reproducibility of strain-induced modulation of the nonlinear optical response in this material.

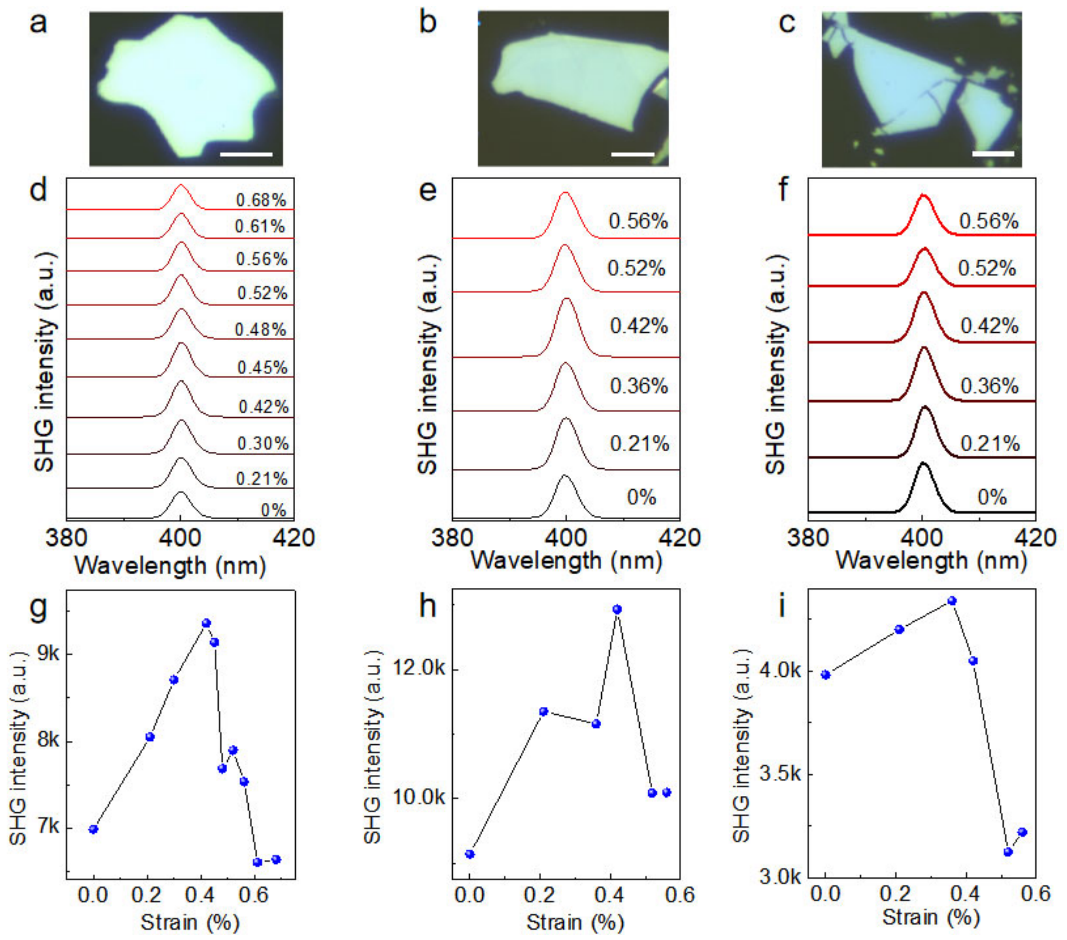

Figure S4. Reproducibility of the strain-dependent SHG modulation in few-layer ε-InSe: (a–c) Bright-field optical microscopy images of three representative samples selected for the strain-dependent measurements. (d–f) Evolution of the SHG spectra with increasing tensile strain for the three samples, respectively. (g–i) Extracted SHG intensity as a function of applied tensile strain, derived from the spectra shown in (d–f).

To confirm the reversibility and reproducibility of the strain-dependent SHG response, we carried out loading–unloading–reloading cycle measurements on a single few-layer ε-InSe flake (Fig. S5). In the first loading cycle (Fig. S5 a, b), the SHG intensity increases with tensile strain up to ~0.42% and then decreases upon further straining. After complete strain release, a second loading cycle was applied to the same flake (Fig. S5 c, d), yielding an SHG intensity evolution that is nearly identical to that of the first cycle. These results clearly demonstrate that the strain-induced SHG modulation is fully reversible and reproducible, indicating that the lattice deformation is elastic and the strain transfer remains stable without stress relaxation or interfacial slippage. The observed reproducibility and cyclic stability confirm the intrinsic nature of the non-monotonic strain-tuning behavior in ε-InSe and support its potential for reversible and reusable flexible optoelectronic applications.

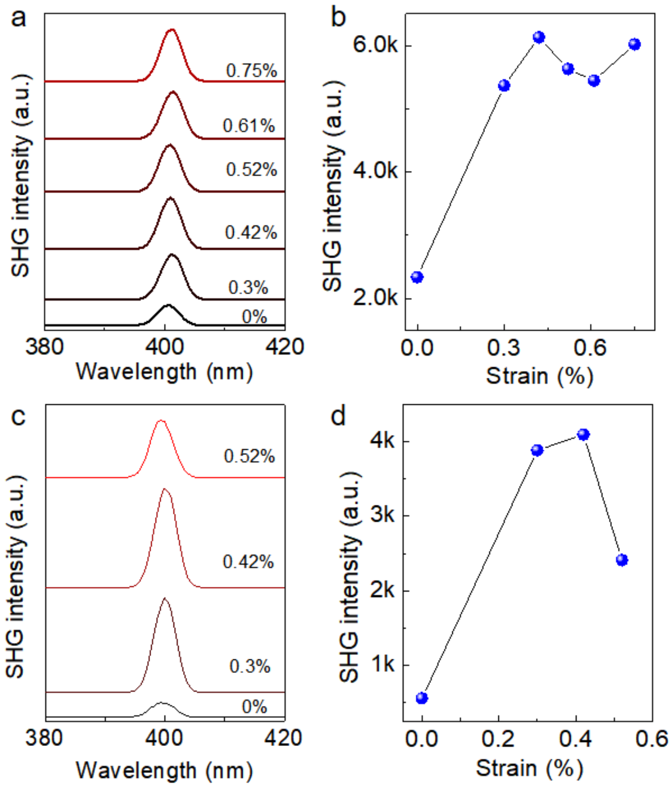

Figure S5. Cyclic loading test of strain-dependent SHG in few-layer ε-InSe: (a, b) SHG spectral evolution and extracted intensity as a function of strain during the initial straining process. (c, d) Corresponding data obtained after strain release and reapplication, showing the reversibility of the strain response.

To further verify the spatial uniformity of the strain-induced SHG modulation, we conducted SHG intensity mapping on another few-layer ε-InSe flake under uniaxial tensile strain. The optical micrograph of the PVA-encapsulated flake is shown in Fig. S6a, revealing a flat and well-defined geometry. Upon application of 0.42% tensile strain (Fig. S6 c), a significant enhancement of the SHG intensity is observed over the entire flake, suggesting that the strain-induced lattice distortion effectively enhances the second-order susceptibility $\chi^{(2)}$. At a higher strain of 0.52% (Fig. S6 d), however, the SHG intensity decreases while still exceeding its initial value. The SHG mapping consistently displays a non-monotonic evolution, and the intensity distribution remains spatially homogeneous throughout the straining process, confirming the efficient and uniform strain transfer to the ε-InSe flake.

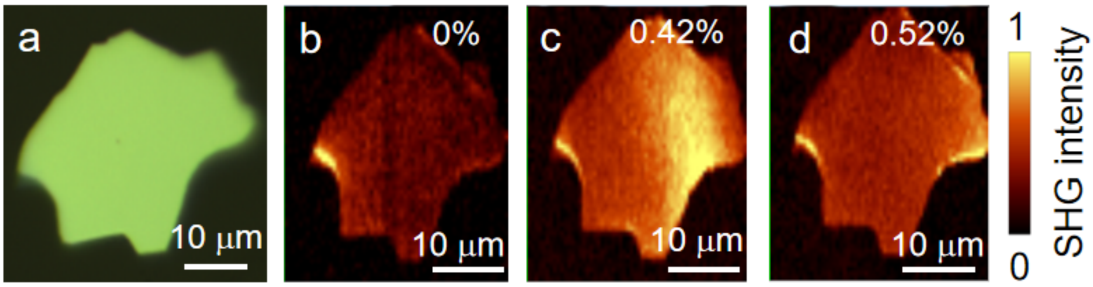

Figure S6. Spatial mapping of strain-dependent SHG in few-layer ε-InSe: (a) Optical micrograph of a PVA-encapsulated few-layer ε-InSe flake. (b–d) Normalized SHG intensity distribution maps acquired on the same flake under strain-free conditions (b), at 0.42% tensile strain (c), and at 0.52% tensile strain (d). Scale bars: 10 μm.

Note 5. Strain-Tunable PL spectroscopy

To verify the effective modulation of the ε-InSe bandgap by strain, we performed PL spectroscopy on few-layer ε-InSe (about 31 nm) under uniaxial tensile strain. As shown in Figure S7a, with increasing tensile strain from 0 to 0.72%, the PL peak redshifts from approximately 1.29 eV to 1.23 eV, corresponding to a total shift of about 60 meV. This redshift reflects the efficient strain-induced tuning of the ε-InSe band structure. The in-plane tensile strain increases the In–Se bond length, weakening the hybridization strength between atomic orbitals and resulting in a downward shift in the conduction band minimum and an upward shift in the valence band maximum, thereby narrowing the bandgap [8]. The peak position exhibits a good linear dependence on the strain, with a shift rate of approximately 73 meV/% strain (Figure S4b), which is consistent with previously reported values [8] and demonstrates the high strain sensitivity of ε-InSe.

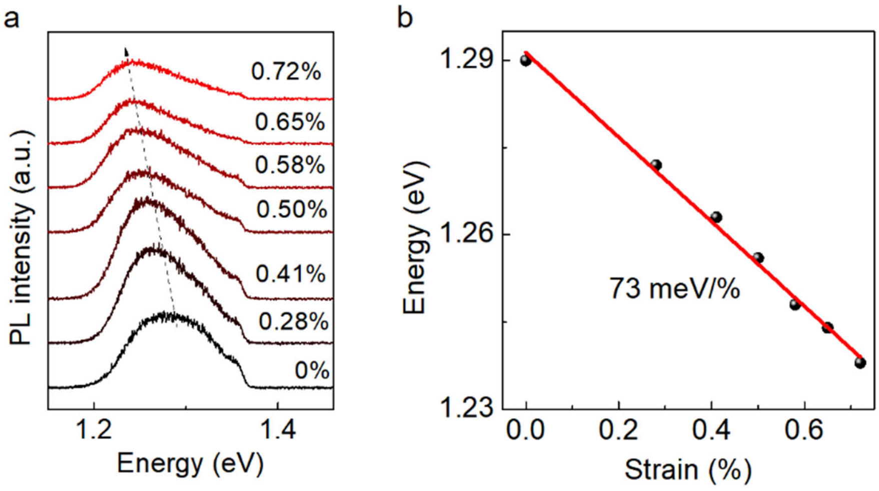

Figure S7. Strain-dependent PL spectra of ε-InSe: (a) PL spectra of a 31 nm thick ε-InSe flake under increasing tensile strain. (b) Extracted PL peak energy as a function of applied strain; the red line is a linear fit to the data, yielding the strain-induced tuning rate.

References

1. Shubina, T. V.; Desrat, W.; Moret, M.; Tiberj, A.; Briot, O.; Davydov, V. Y.; Platonov, A. V.; Semina, M. A.; Gil, B., InSe as a case between 3D and 2D layered crystals for excitons. *Nat. Commun.* **2019,** *10* (1), 3479.
2. Bandurin, D. A.; Tyurnina, A. V.; Yu, G. L.; Mishchenko, A.; Zólyomi, V.; Morozov, S. V.; Kumar, R. K.; Gorbachev, R. V.; Kudrynskyi, Z. R.; Pezzini, S.; Kovalyuk, Z. D.; Zeitler, U.; Novoselov, K. S.; Patanè, A.; Eaves, L.; Grigorieva, I. V.; Fal'ko, V. I.; Geim, A. K.; Cao, Y., High electron mobility, quantum Hall effect and anomalous optical response in atomically thin InSe. *Nat. Nanotechnol.* **2017,** *12* (3), 223-227.
3. Choy, M. M.; Byer, R. L., Accurate second-order susceptibility measurements of visible and infrared nonlinear crystals. *Phys. Rev. B* **1976,** *14* (4), 1693-1706.
4. Zhou, J.; Shi, J.; Zeng, Q.; Chen, Y.; Niu, L.; Liu, F.; Yu, T.; Suenaga, K.; Liu, X.; Lin, J.; Liu, Z., InSe monolayer: synthesis, structure and ultra-high second-harmonic generation. *2D Mater.* 2018, 5 (2), 025019.
5. Janisch, C.; Wang, Y.; Ma, D.; Mehta, N.; Elías, A. L.; Perea-López, N.; Terrones, M.; Crespi, V.; Liu, Z., Extraordinary Second Harmonic Generation in Tungsten Disulfide Monolayers. *Sci. Rep.* 2014, 4 (1), 5530.
6. Bredillet, K.; Riporto, J.; Forcherio, G. T.; Dunklin, J. R.; Wolf, J.-P.; Bonacina, L.; Mugnier, Y.; Le Dantec, R., Dispersion of the nonlinear susceptibility of MoS_2 and WS_2 from second-harmonic scattering spectroscopy. *Phys. Rev. B* 2020, 102 (23), 235408.
7. Zhou, X.; Cheng, J.; Zhou, Y.; Cao, T.; Hong, H.; Liao, Z.; Wu, S.; Peng, H.; Liu, K.; Yu, D., Strong Second-Harmonic Generation in Atomic Layered GaSe. *J. Am. Chem. Soc.* 2015, 137 (25), 7994-7997.
8. Song, C.; Fan, F.; Xuan, N.; Huang, S.; Zhang, G.; Wang, C.; Sun, Z.; Wu, H.; Yan, H., Largely Tunable Band Structures of Few-Layer InSe by Uniaxial Strain. *ACS Appl. Mater. Interfaces* **2018,** *10* (4), 3994-4000.
